# Dr. Bright on Dropsical Effusion from Liver Disease

**Published:** 1828-04-01

**Authors:** 


### Medico-Chirurgical Review,

### No. XVI.

[FASCICULUS II.]

JANUARY 26, 1828.

### Art. III.

### *Cases illustrative of some of the Appearances observable after Death, when* Dropsical Effusion *has been connected with Disease of the Liver.* By Richard Bright, M.D.

[Reports, &c. from Guy's Hospital.]

"Quum vero a splene aut hepate in hydropem transitus fit, effugiunt non valde."—Hippocrates, *de Affectionibus, lib.* xiv. *cap.* xxiii.

"Neque ignoro, Erasistrato displicuisse hanc curandi viam: morbum enim hunc *jocinoris* putavit: ita illum esse sanandum."—Celsus, *lib.* iii. 21.

"Corrupti jecoris vitio vel splenis acerbus crescit hydrops."—Serenus Samonicus.

THE three quotations which we have introduced from three of the most ancient works on medicine, will shew at what an early period dropsy was traced to disease of the liver. Erasistratus, like some hobby-horse riders of modern times, wrote a book to prove that dropsy was *always* dependent on hepatic disease; but the remark of Celsus on this doctrine is equally terse and true. "Sed primum, non hujus visceris unius hoc vitium (hydrops) est: nam et liene affecto, et in totius corporis malo habitu fit." Loco citato. We shall not, however, wade through the innumerable speculations which have been entertained as to the nature, causes, or treatment of the different species of dropsy. We shall only remark one or two curious facts; first, that the father of physic recognized certain cases of anasarca which occurred suddenly in robust constitutions, and required blood-letting.—*De Victu Acutorum*, lxii. How far Hippocrates anticipated modern pathologists on this point, it is not necessary to inquire. The second remark is, that Areteus appears first to have noticed hydatid—or rather ovarian dropsy, in which paracentesis was not effectual. This sagacious disciple of Esculapius made a confession, which, though unpalatable to the "march of intellect" in the present day, contains, nevertheless, some truth. He tells us that few have been cured of dropsy—and that those who have been so fortunate as to escape, may thank the Gods, rather than the doctors, for their recovery!

But we must come at once to the work before us. In our last number, we gave an analysis of Dr. Bright's first division, in which an attempt was made to prove that many cases of dropsy depended on certain organic changes in the kidneys themselves. The next division relates to the etiology of dropsy, as connected with hepatic disease. Dr. B. sets out by expressing a conviction, that many cases of this effusion are owing to renal, which are set down to the account of hepatic affection—but, at the same time, he entertains no doubt that, "in many other cases, the liver is the real cause of the dropsical effusion, frequently shewing most extensive disease, when the kidneys are quite healthy." In fact, it did not escape Dr. Bright's notice—and it cannot escape the notice of his readers, that, in almost every case which he has detailed of *renal* disease, in connexion with dropsy, there was also more or less of disease in the liver. The dissections of Morgagni, Bonetus, and Lieutaud, corroborate the facts disclosed by Dr. Bright, and all these circumstances, taken in connexion, tend to throw a considerable shade of doubt on Dr. Bright's doctrine of the renal origin of dropsy. The same doubt, however, does not apply to the present section of the work under review.

In the preceding section, Dr. B. had often expressed himself thus:—"The liver shewed a tendency to granulation"—thus intimating a doubt as to the existence of actual change of structure. Dr. B. explains himself in the following terms.

"The fact is, that the liver in these cases has usually preserved its natural figure; the acute margin has been perfect, and the general size has not been augmented; the peritoneum has been quite transparent, and attached only in the ordinary degree to the viscus; the texture of the liver has neither been unnaturally firm nor morbidly flaccid; but, on examining the surface, it has been evident that the colour was less uniform than in perfect health: the whole was marbled, consisting of very small light spots in a darker ground; but on making a section perpendicular to the surface, though the same general variety of colour has been observed, yet in some parts of the section it has been doubtful whether the darker or the lighter part should be considered as the ground-work: in general however, by attentive observation, it will be found that, in the centre of the lighter spots, small depressions or openings are visible, and that the darker parts appear to be the connecting medium of the lighter parts, which seem to be the acini of the glandular structure. Although in most cases these appearances scarcely attract attention, yet in other cases they become more obvious, either the white portions becoming larger in proportion, or the whole viscus appearing to have lost a little of its natural pliability, to have become hard, and to break down with a slightly granulated fracture."

In all these cases, Dr. B. observes, "the secretion of the bile is tolerably natural, the gall-bladder being well supplied with bile, of a sufficiently dark yellow colour." We have reason to believe, that much error has arisen respecting the healthy secretion of the liver, because the colour of the bile has appeared natural in the gall-bladder and in the secretions. It should be recollected that it is in the gall-bladder the bile assumes that colour which tinges the fæces—and that only a certain portion of the bile that passes into the intestines has ever been in the gall-gladder. Now the biliary secretion may be much changed, and yet that portion of it which regurgitates into the above-mentioned reservoir may there assume the *usual colour*, while greatly deficient in its other qualities. We must not, therefore, be guided entirely by the colour of the motions, but by the smell, and other properties. How often do we find the secretions unbearably fetid, while their colour is brown, or even yellow? The states of the digestion, the complexion, nay, the urine itself, are all influenced by the state of the biliary organ.

Besides the appearances above described by Dr. Bright, in the livers of dropsical subjects, he has occasionally seen the organ deviating in its consistence from the natural state, "being either too firm or too flaccid," though such changes are doubtless seen where no dropsical effusions obtain. From the prominent place which the renal disease appeared to hold in these cases, our author was induced to consider the hepatic derangement as secondary or subordinate, "though not impossibly the state of both these organs depends on the same general constitutional affection; and I have sometimes even thought that the tendency to granulation, where it existed, maintained a certain relation, in its progress, to the disease in the kidney." Moreover, the author justly remarks, that there are hepatic derangements, unaccompanied by obvious disease in other organs, which may probably be, with propriety, considered as laying the foundation of dropsical effusions. To a detail of cases of this kind Dr. Bright next proceeds, and we shall follow him.

*Case* 1. W. Taylor, aged 66, by profession an architectural drawer, but now a pauper, was admitted on the 4th January, 1826. He confessed that he had lived hard, and it was evident that reverses of fortune and disappointed hopes, might have injured his *morale*. For the four preceding months, his appetite had failed, and still more recently his legs began to swell; his abdomen became tumid, and his flesh wasted. His countenance was sallow, conjunctivæ slightly yellow, bowels relaxed, pulse weak, abdomen tender on pressure, especially in the region of the liver—and altogether he had the appearance of being completely broken down. The motions did not seem deficient in bile, nor unnatural in colour; but the urine was loaded with a pink sediment, and was not coagulable by heat. We need not give the details of 16 days' unsuccessful practice. On the 20th January he sat up, and seemed more revived. In the evening he was seized with dyspnœa, and died in the night.

*Dissection.* No effusion into the cavities of the pleura—very little of the lungs was crepitous; yet they were not hepatized nor tuberculated. They were congested, and loaded with serum—in short, they were œdematous. Heart sound—abdomen contained seven or eight pounds of clear straw-coloured serum—body and limbs œdematous. The intestines unaffected. Liver contracted, and of a morbid structure throughout, apparently from depositions of minute portions of yellow matter. The surface presented a general rough granular feel, the colour being a liver red and yellowish gray. The same structure pervaded the whole of the interior. The liver was, upon the whole, smaller than natural, and broke down easily with a brittle crisp fracture, uneven and granular. The gall-bladder was opake and thick, containing the usual quantity of bile. The orifice of the ductus communis was contracted into a nipple-like projection, with an orifice not larger than to admit a pin. The gall-bladder contained a deep-coloured viscid bile, and, when emptied, presented, on its internal surface, a number of minute yellow bodies, rather larger than millet-seed, and soft. The pancreas was soft—spleen very small—the kidneys smaller than natural, but perfectly healthy.

In the above case, from the appearances on dissection, Dr. Bright was led to suspect, that part of the structural change in the liver might depend on some deposit from the bile similar to that which obtained on the inner surface of the gall-bladder. Dr. Bostock was, therefore, applied to, for the purpose of chemically examining into this point. Dr. B.'s chemical processes are given, in a letter from that gentleman; but we shall content ourselves with the results.

"From the above observations I think we are warranted in concluding, that the liver which you sent me for examination contained a quantity of a substance nearly resembling cholesterine, the body which forms the basis of the biliary calculi. I do not venture to determine concerning the nature of the connexion which subsisted between this substance and the liver, but I should conjecture that it had been secreted by the arteries of this organ, and deposited in its cellular texture."

*Case* 2. J. Macdonald, a youth of 15, was admitted on the 21st of June, 1826. He was of weakly constitution, but said he had enjoyed good health till within two months. At that time his legs began to swell, and latterly his abdomen. It was now considerably enlarged, and a tumour could be distinctly felt in the region of the liver. The legs were slightly œdematous—emaciation general—urine scanty, and *not* coagulable. Diuretics and mercurials were given, and some slight improvement ensued; but, about the beginning of September, the boy became evidently worse, and on the 27th of that month he was tapped, when three gallons of straw-coloured serum were drawn off. On the 1st of October, the tumour of the liver is reported to be felt completely tuberculated. He died exhausted on the 16th October.

*Dissection*. There were slight marks of peritoneal inflammation, and some flakes of coagulable lymph in the abdominal effusion. The liver was externally tuberculous, of a light yellow colour, with deep fissures on its surface, apparently arising from partial contractions in the substance of the organ, or its adventitious investing membrane. The liver, which was about one third larger than natural, was also increased in density and specific gravity, cutting with considerably more resistance than boiled udder, to which it bore some resemblance. Its whole structure was composed of bright yellow granules, distributed in a transparent pinkish ground, the two parts bearing nearly an equal proportion. There was no appearance of tubercular structure in the organ. The gall-bladder was contracted, containing a small quantity of dirty-looking bile—kidneys rather pale, with irregular vascularity—lungs and heart quite healthy.

*Case* 3. Thomas Holbeach, aged 60, was admitted on the 12th October, 1825, in a lamentable state of dropsy. His whole body was unwieldy—his legs and thighs greatly swollen, with ill-conditioned ulcers on his shins. He lies propped up in bed, continually moaning. Urine is scanty and rather high-coloured—motions frequently loose, but not very deficient in bile—tongue dry and brown. He was ordered squills, blue-pill, and opium, with some diuretics; but although he sometimes shewed symptoms of amelioration, he sunk exhausted on the 23d November, about five weeks after he came to the hospital.

*Dissection.* On opening the abdomen a singular appearance presented itself to view, when the water was drawn off. All the viscera stood rigidly raised like rock-work. The liver formed two whitish flesh-coloured masses, the edges thickened and rounded, and the whole surface somewhat tuberculous. Below the liver, to the left, was an irregular mass, purplish in colour. It was found to be a mass of omentum and colon matted together by an adventitious membrane, which appeared to cover the whole. Below this were seen four or five convolutions of intestine perfectly erect and stiff, of a purplish green or livid colour, covered by the same adventitious membrane. These convolutions felt thick, hard, and elastic. The substance of the liver was found hardened throughout, the structure nearly resembling scirrhus, with bands of thickened cellular membrane, like ligamentous matter pervading every part—in some places forming one third of the whole structure. There were no tubercles in the interior of the organ, which felt nearly as hard as cartilage. There were old adhesions between the liver and diaphragm. The gall-bladder was contracted, and covered by false membrane, and contained bright yellow bile, the ducts being pervious. The coats of the intestines were, in some places, the sixth of an inch in thickness. The kidneys were healthy —the lungs, in some places, œdematous and flabby, but, on the whole, not unhealthy. There was some effusion into the cavities of the thorax and pericardium.

Having thus given three out of the seven cases detailed by our author, in illustration of his subject, we do not deem it necessary to notice any more.

Dr. Bright observes that the foregoing cases present at least three distinct morbid conditions of the liver, all terminating in dropsical effusion into the abdomen. Thus, in one case, (W. Taylor) a distinct morbid deposit, or a conversion of matter had taken place around or in the secreting portion of the organ, which, without interfering with the natural consistence of the liver, rendered its surface rough, and its whole texture deranged and granular.

In another case, (No. 2, Macdonald) both the secreting part and the connecting cellular tissue of the liver had suffered a change of structure nearly in an equal degree, so that the whole viscus was brought to an unusual state of firmness. The acini were enlarged, and the parenchymatous substance was thickened, and brought to a state of semi-cartilaginous hardness, without being drawn into bands.

In a third case, (Holbeach) the diseased state of the cellular membrane seemed to have advanced much further, so that it had formed bands in various directions, not unlike a scirrhous degeneration either in the appearance or the consistency which it assumed. "Yet the secretion in the organ had not been entirely obstructed."

In one case there were no semi-cartilaginous bands of hardened cellular tissue, but the whole organ was changed into globular concretions, harder and more tough than in the natural condition—easily picked out of the cavities in which they were imbedded, and sliding pretty readily over each other, so as to render the whole tough and pliable. In some, there will be found cysts or tubercles—in others a series of abscesses—in short, it would be endless, and moreover useless to enumerate the almost infinite variety of changes which may be seen in diseased livers.

We now proceed to notice Dr. Bright's mode of explaining the way in which the hepatic disease produces the dropsical effusion.

"It appears," says he, "that all of those (organic changes) just now described produce very general obstruction to the circulation through the branches of the vena portæ, and become, in this way, the immediate cause of dropsical effusion, independently of any morbid condition which may result to the blood by its not having given off those substances from which it is purified, while the process of secreting bile is carried on in its full extent. It is these general changes in the structure of the liver which give rise to dropsy, more frequently than any of the circumscribed changes,—as tubercles of various kinds, and hydatids occurring imbedded in the substance; for the influence of these, as long as from their situation they make no immediate pressure on the large vessels, is often very small in favouring serous effusion, however much they may wear out the constitution by the irritation they produce."

For a long time we were in the habit of taking this mechanical view of the production of serous effusions in diseases of the liver; but we have given it up, and believe that it is quite erroneous. That mechanical pressure on veins will produce serous effusions or infiltrations, we admit; but, where is the proof that there is any actual obstruction to the mere flow of blood through the vessels of the liver, in diseased conditions of that organ? In many of the dropsical cases, the organ was not enlarged, but rather diminished, and, consequently, could not press on the contiguous vessels returning blood to the heart—in others, the liver was only triflingly enlarged. Yet we frequently see both the liver and spleen enlarged to an amazing size, without any symptom of dropsy. How many tumours do we find developed in the abdomen, and arrive at an immense growth, which must press upon all the neighbouring vessels, yet without dropsy. These facts did not escape the penetration of the illustrious Bichat, who resolutely denied that dropsical effusions in the abdomen and body generally are to be looked upon as resulting from mechanical obstruction to the passage of blood through the liver—an obstruction which, in fact, has never been shewn to exist. But it will be said that LOWER tied the cava inferior of a dog, and produced dropsy of the abdomen. This was effecting mechanical obstruction with a vengeance! When Dr. Bright, or any who support his doctrine, shew us this obstruction, by injections of the organ, we shall then admit it as the cause of dropsy, but not till then.

It is far more reasonable to conclude that either the causes which produce the liver-disease effect also the dropsical disposition—or, that the altered, and consequently morbid condition of the biliary secretion leads to the serous effusions, by disordering the functions of various organs in the animal economy, including the kidneys, thus vitiating the blood and all other fluids in the body. We have already commented on the fallacy of concluding that bile was healthy if it had the usual colour. The experiments of Dr. Bostock, appended to this section of our author's work, prove that the qualities of the bile were unequivocally deteriorated in the various specimens examined. Thus, in one specimen, upon minute inspection, some portions exhibited a yellow tinge, while others were of a light flesh-colour.

"The flesh-coloured part seemed to consist of a dense substance of a uniform texture, while the yellow part appeared to be composed of a number of irregular spots, which gave the peculiar colour to this part imbedded in the dense substance."

In another specimen, Dr. Bostock observes, "the bile was considerably lighter coloured than natural, less viscid, and had a very nauseous odour. It became rapidly putrid, and was then extremely fetid." By chemical examination, he found it to contain a substance that might be considered intermediate between albumen and mucus, "while the resinous or proper biliary matter was much more than ordinary."

In a third specimen, besides other changes, Dr. B. observed "a number of black particles diffused through it, which very slowly subsided." In a fourth specimen, the bile was "unusually thick and tenacious, and of nearly a black colour." It was found to contain a large quantity of a mixture of albumen and mucus. In a fifth specimen, the bile contained a considerable number of biliary calculi, from the size of a pea to that of a grain of sand. In a sixth specimen, the fluid would not have been "recognized as bile," had not Dr. Bright informed Dr. Bostock that it was taken from the gall-bladder. Its consistence was like that of serum, but more tenacious—its odour offensive—its colour a bright light orange. It was neither acid nor alkaline.

Now, in many of the cases from which these specimens of vitiated bile were taken, the motions had a nearly natural *colour*, and hence it was concluded that there could be nothing wrong with the function of the liver, whatever might be the degree of organic change. That this is a great error, we may infer from analogy as well as observation. Do the lungs perform their proper function when disorganized? Do the kidneys secrete healthy urine when their structure is altered? Certainly not. And why should we expect healthy bile (whatever may be the colour) from an unsound liver?

In carefully examining the dissections given by our author, we scarcely find a single instance in which the peritoneal surface was not in a diseased state—either covered with false membranes, or shewing other unequivocal signs of previous inflammation. This fact at once does away with the theory that the dropsical effusion is the result of mere mechanical obstruction to the flow of blood through the liver. The fact is, that the pleura, as well as the peritoneum, takes on a morbid condition in these hepatic diseases, and hydrothorax, ascites, and anasarca, are generally combined.

A perusal of the cases brought forward by Dr. Bright, and a careful observation of facts in actual practice, would lead us to conclude that the structure or function of the liver was disordered, when dropsical effusions and waste of flesh appear in an individual not labouring under any organic disease of the heart or lungs, and who has not been recently subjected to those causes which induce an inflammatory dropsy. To this conclusion we would be induced to come, even if the stools were yellow—seeing, as we have done, that in one of the worst specimens of bile examined by Dr. Bostock, the *colour* of that fluid was a " bright light orange." Now, in such a case, if the alvine secretions were found of the same colour as the hepatic, the practitioner would exclaim—" Oh, here is bile as healthy as that of an infant !" Yet Dr. Bostock says, in his letter, that he should not have recognized it chemically as bile, had not Dr. Bright assured him that he took it from a human gall-bladder ! Such are the fallacies to which the science of medicine is subject. Every path we tread—every step we take—every indication we act upon, is pregnant with such errors—and the whole practice of medicine, in fact, requires cleansing, as much as did the stable of Augeus.

Tales of error have been hummed into the infant's ear, while rocked in his cradle, or fondled at the breast—they have been engraven on his sensorium and some other parts, at school, by dint of the birch—they have luxuriated into gorgeous forms of classic and philosophic imagery in cloisters and colleges—they have been delivered, ore rotundo, in the assumed garbs of solemn truths and scientific dogmas, in the dissecting room, the class-room, and the clinical ward—they have rolled, and do roll in volumes from the press, with all the impetus and velocity which high-pressure engines and the power of steam can confer—the false notes have been circulated so freely as sterling ore, that nobody thought of examining the water-mark of truth—in short, these tales of error have been more greedily perused than the book of Nature, as fostering at once the indolence of the mind, and the ease of the body ; and, in this way, the field of medicine has become choaked up with weeds that will require many centuries to root out ! We must return again to the paths of Hippocrates and Sydenham—and close observation at the bed-side of sickness must supersede the theories of the closet, and the dreams of the chemical and the mechanical philosophers.

